# Vitamin D deficiency is associated with hepatic decompensation and inflammation in patients with liver cirrhosis: A prospective cohort study

**DOI:** 10.1371/journal.pone.0207162

**Published:** 2018-11-08

**Authors:** Alica Kubesch, Leonie Quenstedt, Maged Saleh, Sabrina Rüschenbaum, Katharina Schwarzkopf, Yolanda Martinez, Christoph Welsch, Stefan Zeuzem, Tania M. Welzel, Christian M. Lange

**Affiliations:** Department of Internal Medicine 1, Goethe-University Hospital Frankfurt, Frankfurt, Germany; Medizinische Fakultat der RWTH Aachen, GERMANY

## Abstract

**Background:**

Vitamin D is required to maintain the integrity of the intestinal barrier and inhibits inflammatory signaling pathways.

**Objective:**

Vitamin D deficiency might be involved in cirrhosis-associated systemic inflammation and risk of hepatic decompensation in patients with liver cirrhosis.

**Methods:**

Outpatients of the Hepatology Unit of the University Hospital Frankfurt with advanced liver fibrosis and cirrhosis were prospectively enrolled. 25-hydroxyvitamin D (25(OH)D_3_) serum concentrations were quantified and associated with markers of systemic inflammation / intestinal bacterial translocation and hepatic decompensation.

**Results:**

A total of 338 patients with advanced liver fibrosis or cirrhosis were included. Of those, 51 patients (15%) were hospitalized due to hepatic decompensation during follow-up. Overall, 72 patients (21%) had severe vitamin D deficiency. However, patients receiving vitamin D supplements had significantly higher 25(OH)D_3_ serum levels compared to patients without supplements (37 ng/mL vs. 16 ng/ml, P<0.0001). Uni- and multivariate analyses revealed an independent association of severe vitamin D deficiency with the risk of hepatic decompensation during follow-up (multivariate *P* = 0.012; OR = 3.25, 95% CI = 1.30–8.2), together with MELD score, low hemoglobin concentration, low coffee consumption, and presence of diabetes. Of note, serum levels of C-reactive protein, IL-6 and soluble CD14 were significantly higher in patients with *versus* without severe vitamin D deficiency, and serum levels of soluble CD14 levels declined in patients with *de novo* supplementation of vitamin D (median 2.15 *vs*. 1.87 ng/mL, P = 0.002).

**Conclusions:**

In this prospective cohort study, baseline vitamin D levels were inversely associated with liver-cirrhosis related systemic inflammation and the risk of hepatic decompensation.

## Introduction

Vitamin D is a key hormone in the regulation of bone metabolism [1]. However, vitamin D receptor (VDR) signaling has been shown to impact on the expression of more than 9000 genes, and accumulating evidence suggests that these genomic effects of calcitriol, the bioactive form of vitamin D, execute important non-classical (in contrast to bone metabolism) functions of vitamin D such as regulation of immune responses or cell proliferation [2]. For example, vitamin D serves as an anti-inflammatory mediator by inhibiting the differentiation of Th17 cells in in favor of regulatory T cells [3]. Furthermore, it was shown that supplementation with the vitamin D precursor cholecalciferol results in a decrease of C-reactive protein (CRP), IL-6 and TNF-α serum levels in hemodialysis patients [4]. A significant reduction of systemic inflammation and consecutive resolution of inflammation has also been observed in an interventional study with high-dose cholecalciferol in patients with lung tuberculosis [5].

Additional studies have suggested a role of VDR signaling in maintaining the integrity of the intestinal barrier and intestinal bacterial homeostasis. For example, a large prospective study has shown that baseline vitamin D deficiency is associated with an increased risk of inflammatory bowel disease during follow-up [6]. Mechanistically, this might be explained by direct effects of VDR signaling on tight junctions and integrity of intestinal epithelial cells [7,8]. Furthermore, polymorphisms in the VDR were identified by a genome-wide association study to be associated with distinct composition of the gut microbiota [9].

Both, systemic inflammation and dysfunction of the intestinal barrier are key drivers of the progression of chronic liver disease. Since vitamin D deficiency is particular frequent in patients with liver disease such as viral hepatitis, or alcoholic hepatitis [10–14], we aimed to further explore the relationship between vitamin D serum levels, systemic inflammation and hepatic decompensation in a prospective cohort study of patients with liver cirrhosis.

## Patients and methods

### Study population

Since July 2016, consecutive patients presenting to the outpatient liver clinic of the University Hospital Frankfurt, Germany, with advanced liver fibrosis or cirrhosis were included in a prospective cohort study. Inclusion criteria were age above 18 years, diagnosed advanced fibrosis (Metavir ≥F2) or cirrhosis of different etiologies, and written informed consent to participate in the study. Exclusion criteria were age below 18 years, pregnancy or breastfeeding, presence of hepatocellular carcinoma (HCC) beyond Milan criteria, presence of infection with human immunodeficiency virus (HIV), or therapy with immunosuppressive agents. The patients were followed up every three months and when admitted to hospital with hepatic decompensation. At each follow-up time point, clinical and laboratory variables including 25-hydroxyvitamin D concentration were recorded and serum samples were stored for further analyses. Vitamin D supplementation was initiated in a number of patients according to the responsible physicians’ discretion.

Liver fibrosis was assessed by shear-wave elastography using a Hitachi HI VISION Ascendus system (pSWE (ARFI); thresholds F3 = >9.5 kPa, F4 = >12.5 kPa) or a Siemens Acuson S2000^TM^ system (pSWE (ARFI) Virtual Touch Quantification (VTQ); thresholds F3 = >1.55 m/s, F4 = >1.8 m/s). In addition, liver fibrosis was assessed by the FibroTest^TM^. Acute decompensation of liver cirrhosis was defined as presence of one of the following criteria: new onset / progression of hepatic encephalopathy graded by West-Haven criteria [15], gastrointestinal hemorrhage, bacterial infection, or ascites grade II-III (graded according to *Moore et al*.[16]).

The study was approved by the ethics committee of the University- Hospital Frankfurt prior to the study. A written informed consent was obtained from the patients prior to enrollment in the study.

### Quantification of 25-hydroxyvitamin D [25(OH)D_3_] serum levels

25-hydroxyvitamin (25(OH)D_3_) levels were quantified by radioimmunoassay as previously described [17]. 25(OH)D_3_ levels above >30 ng/ml were considered as optimal, whereas 25(OH)D_3_ levels between 10–30 ng/ml and <10 ng/ml were considered as deficiency and severe deficiency, respectively.

### Quantification of IL-6, I-FABP, and soluble CD14 serum levels

Serum concentrations of IL-6, I-FABP, and soluble CD14 were quantified by ELISA as described previously [18].

### Statistical analyses

Statistical analyses were performed using BiAS, Version 11.06, and Graphpad PRISM5. Group differences were assessed by means of χ^2^ contingency tables or Wilcoxon-Mann-Whitney-U-tests, as appropriate. *P* values < 0.05 were considered to be statistically significant. Associations of outcomes with continuous or dichotomic variables were assessed in linear and logistic regression models, respectively. After univariate analyses, multivariate analyses were performed for significant associations. Multivariate models were obtained by backward selection, using a *P* value >0.15 for removal from the model.

## Results

### Patient characteristics

A total of 338 patients with advanced liver fibrosis or cirrhosis of different etiologies were recruited for the present study according to the above defined inclusion criteria. Baseline characteristics of included patients are shown in Table 1. During a median follow-up time of 3–12 months, 51 out of 338 patients (15%) were hospitalized due to hepatic decompensation, including 23 episodes of acute ascites, 16 episodes of acute hepatic encephalopathy, 7 infections, 16 cases of renal failure, and 6 cases of upper GI bleeding. Of patients who were hospitalized with hepatic decompensation, 36 and 44 had a previous history of ascites and overall hepatic decompensation, respectively, whereas 105 and 130 patients without hospitalization at follow-up had a previous history of ascites and overall hepatic decompensation, respectively. Sixteen patients (5%) died during follow-up.

**Table 1:** Baseline characteristics of included patients.

	No Hospitalization due to decompensation at follow-up N = 287	Hospitalization at follow-up due to decompensation N = 51
Age (years); [SD]	59.40 [12.09]	61.45 [10.99]
Coffee (cups/day); [SD]	1.65 [1.65]	0.47 [0.92]
Diabetes (presence); N [%]	63 [22]	21 [41]
BMI (kg/m^2^); [SD]	27.10 [4.95]	26.20 [5.81]
MELD Score; [SD]	9.68 [3.83]	13.76 [4.39]
Creatinin (mg/dl); [SD]	0.86 [0.29]	1.14 [0.83]
Albumin (g/dl); [SD]	4.12 [0.62]	3.25 [0.59]
Bilirubin (mg/dl); [SD]	1.33 [1.45]	2.27 [1.68]
ALT (U/L); [SD]	37.59 [43.70]	34.62 [24.60]
yGT (U/L); [SD]	111.3 [144.82]	205.66 [296.35]
Leukocyte (/nl); [SD]	6.03 [2.23]	5.66 [2.74]
Hb (mg/dl); [SD]	13.42 [1.91]	11.15 [2.31]
Platelets (/nl); [SD]	141.05 [71.21]	122.96 [78.65]
25(OH)D_3_ (ng/ml); [SD]	27.95 [17.81]	21.24 [18.75]
IL-6 (pg/mL); [SD]	3.61 [4.93]	1.52 [34.03]
I-FABP (pg/mL);[SD]	1155.5 [928.16]	1973.2 [2050.9]
sCD14 (ng/mL); [SD]	2.15 [0.66]	2.58 [0.59]
Ascites at baseline, N [%]	26 [9]	27 [52]
Hepatic encephalopathy at baseline, N [%]	1 [0.35]	2 [4]
History of ascites, N [%]	105 [37]	36 [71]
History of hepatic decompensation, N [%]	13 [45]	44 [86]
**Liver cirrhosis etiology of entire cohort n,(%)**		
HCV 117 (35)		
HBV 53 (16)		
Alcoholic 103 (30)		
NASH 33 (10)		
Other 32 (11)		

ALT, alanine aminotransferase; BMI, body mass index; range; γGT, γ-glutamyl transferase; Hb, hemoglobin; I-FABP, intestinal fatty acid binding protein; MELD, model of end-stage liver disease.

### 25(OH)D_3_ serum levels

The median 25(OH)D_3_ serum concentration of the entire cohort was 23 ng/mL. Of note, 118 patients (35%) out of the entire cohort (n = 338) received vitamin D supplements (1000 IU cholecalciferol daily in 56 patients, 20000 IU cholecalciferol once weekly in 62 patients). The median 25(OH)D_3_ serum concentration was significantly higher in patients receiving vitamin D supplements compared to patients who were not supplemented (37 ng/mL vs. 16 ng/ml, P<0.0001). Of the entire cohort, 132 patients (39%) had optimal 25(OH)D_3_ serum levels >30 ng/ml, whereas 72 patients (21%) had severe vitamin D deficiency (25(OH)D_3_ serum concentration <10 ng/ml). Only 3 out of 72 patients (4%) with severe vitamin D deficiency received vitamin D supplements (more details in Table 2).

**Table 2:** 25(OH)D3 serum levels in patients with advanced liver fibrosis/cirrhosis.

25(OH)D_3_ serum levels (ng/mL)	
**Overall (N = 338)**	
25(OH)D_3_ >30 ng/ml, N [%]	132 [39]
25(OH)D_3_ 30–20 ng/ml, N [%]	61 [18]
25(OH)D_3_ 20–10 ng/ml, N [%]	73 [22]
25(OH)D_3_ <10 ng/ml, N [%]	72 [21]
**Patients on vitamin D supplements (N = 118**)	
25(OH)D_3_ >30 ng/ml, N [%]	84 [71]
25(OH)D_3_ 30–20 ng/ml, N [%]	24 [20]
25(OH)D_3_ 20–10 ng/ml, N [%]	7 [6]
25(OH)D_3_ <10 ng/ml, N [%]	3 [3]
**Patients without vitamin D supplements (N = 220)**	
25(OH)D_3_ >30 ng/ml N; [%]	48 [22]
25(OH)D_3_ 30–20 ng/ml N; [%]	37 [17]
25(OH)D_3_ 20–10 ng/ml N; [%]	66 [30]
25(OH)D_3_ <10 ng/ml N; [%]	69 [31]

Seasonal variations of 25(OH)D_3_ serum levels are shown in S1 Table. Of note, seasonal variations of 25(OH)D_3_ serum were extensive in patients not receiving vitamin D supplements, whereas vitamin D supplementation strongly attenuated seasonal fluctuations of 25(OH)D_3_ serum levels.

Next, multivariate linear regression analyses were performed to explore association with vitamin D serum levels in patients with advanced liver fibrosis or cirrhosis. The analyses were conducted separately for the entire cohort as well as for the subgroup of patients with and without vitamin D substitution. As shown in Table 3, waist circumference, smoking, and history of ascites were independently associated with lower 25(OH)D_3_ serum levels, whereas no significant association between 25(OH)D_3_ serum levels and MELD score, coffee or alcohol consumption were observed in the entire cohort. These results were largely comparable in the subgroups of patients with or without vitamin D substitution (Table 3).

**Table 3:** Linear regression analyses of 25(OH)D_3_ serum levels in patients with advanced liver fibrosis/cirrhosis.

	Univariate analysis		Multivariate analysis	
	*P*-value	beta (SD beta)	*P*-value	beta (SD beta)
**Entire Cohort**				
Waist circumference (cm)	0.006	-0.25 (0.09)	0.005	-0.24 (0.08)
Smoking	0.005	-8.62 (3.01)	0.0008	-9.28 (2.71)
Alcohol consumption (>20g/d)	0.17	-4.6 (3.38)		
Coffee (cups / day)	0.7	0.33 (0.91)		
MELD score	0.5	0.21 (0.34)		
GPT (U/mL)	0.7	-0.01 (0.03)		
gGT (U/mL)	0.5	-0.004 (0.01)		
Shear-wave elastography (m/s)	0.07	0.02 (0.06)		
Presence/history of ascites	0.002	-13.08 (4.16)	0.001	-12.51 (3.77)
**Subgroup of patients with vitamin D supplementation**				
Waist circumference (cm)	0.03	-0.31 (0.14)	0.02	-0.29 (0.13)
Smoking	0.10	-7.33 (4.44)	0.1	-6.44 (3.99)
Alcohol consumption (>20g/d)	0.5	8.35 (12.97)		
Coffee (cups / day)	0.7	0.51 (1.50)		
MELD score	0.6	-0.25 (0.54)		
GPT (U/mL)	0.8	-0.01 (0.03)		
gGT (U/mL)	0.9	6.92 (0.01)		
Shear-wave elastography (m/s)	0.1	4.91 (2.61)	0.01	3.83 (2.29)
Presence/history of ascites	0.6	-3.24 (7.43)	0.001	-12.51 (3.77)
**Subgroup of patients without vitamin D supplementation**				
Waist circumference (cm)	0.3	-0.09 (0.08)		
Smoking	0.002	-9.38 (2.94)	0.0002	-10.55 (2.78)
Alcohol consumption (>20g/d)	0.8	-0.79 (12.97)		
Coffee (cups / day)	0.7	0.35 (0.85)		
MELD score	0.25	0.39 (0.34)		
GPT (U/mL)	0.25	-0.04 (0.03)		
gGT (U/mL)	0.4	-0.005 (0.006)		
Shear-wave elastography (m/s)	0.4	1.27 (1.39)	0.01	3.83 (2.29)
Presence/history of ascites	0.001	-13.1 (4.00)	0.00008	-12.36 (3.59)

ALT, alanine aminotransferase; γGT, γ-glutamyl transferase; MELD, model of end-stage liver disease.

### Baseline vitamin D deficiency is associated with hepatic decompensation at follow-up

**Fig 1:**
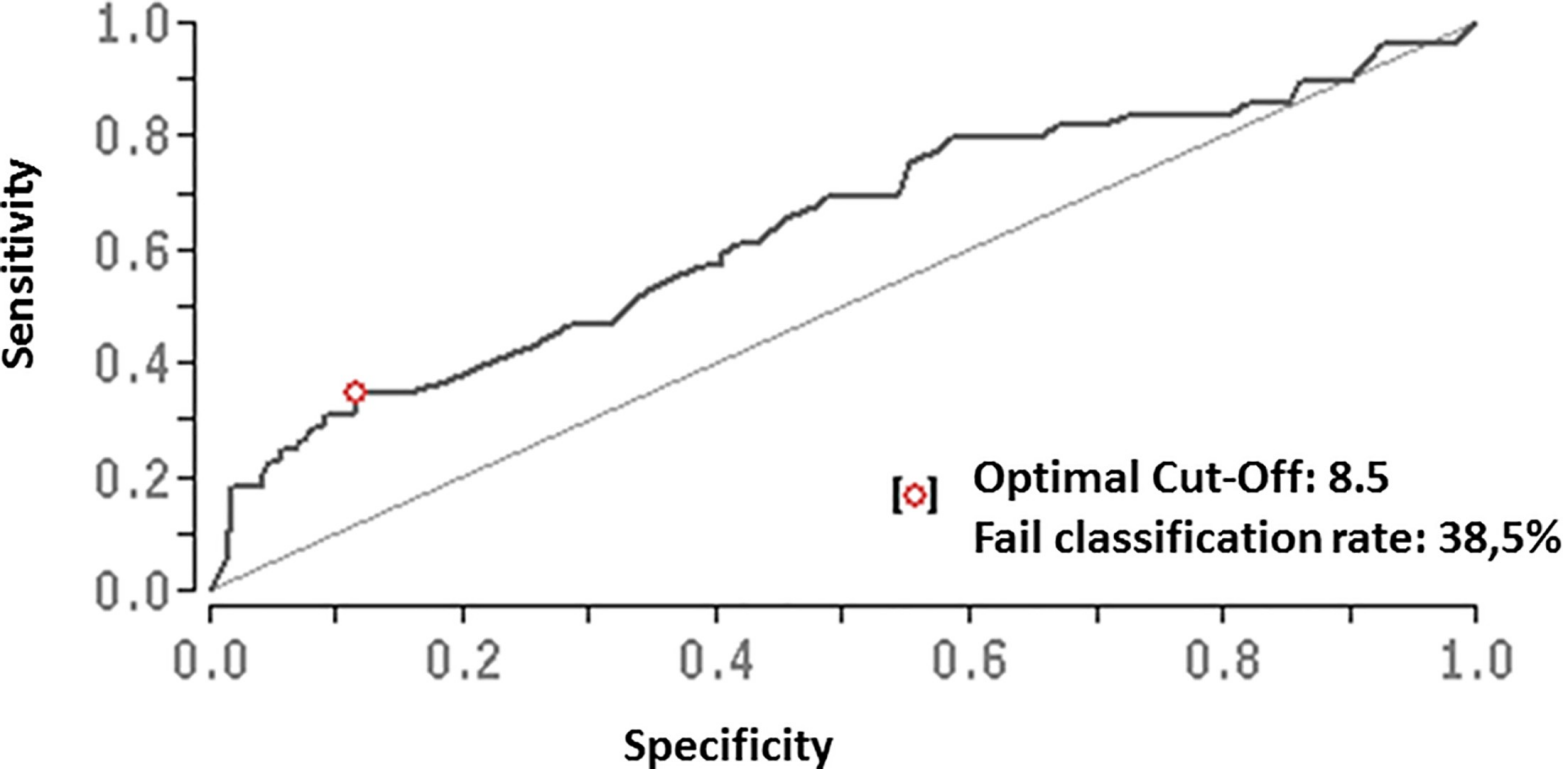
Area under the receiver operating characteristic curve (AUROC) of baseline vitamin D serum levels predicting the risk of hepatic decompensation during follow-up. A 25(OH)D_3_ serum concentration of 8.5 ng/mL was identified as the optimal cutoff.

Uni- and multivariate analyses revealed an independent association of severe vitamin D deficiency with the risk of hepatic decompensation during follow-up (multivariate *P* = 0.012; OR = 3.25, 95% CI = 1.30–8.2), together with MELD score, low hemoglobin concentration, low coffee consumption, and presence of diabetes (Table 4). The association between severe vitamin D deficiency and the risk of hepatic decompensation during follow-up remained significant in the subgroup analysis of patients without vitamin D substitution, but not subgroup analysis of patient with vitamin D substitution (Table 4). Baseline 25(OH)D_3_ serum concentration was also associated with hepatic decompensation during follow-up when assessed as a continuous variable (P = 0.002), or when other cut-offs for vitamin D deficiency were applied (P = 0.01 and P = 0.008 for 25(OH)D_3_ serum concentration <20 ng/mL and <30ng/mL, respectively). Area under the receiver operating characteristic curve (AUROC) revealed a 25(OH)D_3_ serum concentration of 8.5 ng/mL as the optimal cutoff to predict the risk of hepatic decompensation during follow-up (Fig 1). Furthermore, there was a trend for a protective association between vitamin D supplementation and hepatic decompensation (P = 0.1). Finally, severe vitamin D deficiency was associated with mortality during follow-up (P = 0.04), but not with the incidence of hepatocellular carcinoma (P = 0.6).

**Table 4:** Logistic regression analyses of hepatic decompensation in patients with liver cirrhosis.

	Univariate analysis		Multivariate analysis	
	*P*-value	OR (95% CI)	*P*-value	OR (95% CI)
**Entire Cohort**				
Male gender	0.92	0.97 (0.51–1.83)		
Age	0.4	1.01 (0.98–1.04)		
Coffee, cups per day	0.000003	0.36 (0.23–0.55)	0.0002	0.33 (0.18–0.59)
Presence of diabetes	0.004	2.64 (1.37–5.10)	0.001	4.62 (1.86–11.55)
MELD score	<0.00001	1.22 (1.14–1.32)	0.0004	1.20 (1.08–1.32)
Leukocytes / nl	0.2	0.91 (0.79–1.06)		
Hemoglobin (g/dL)	<0.00001	0.62 (0.53–0.73)	0.0004	0.71 (0.59–0.86)
25(OH)D_3_ <10ng/mL	0.01	2.44 (1.22–4.76)	0.01	3.25 (1.30–8.20)
Presence/history of ascites	0.00001	4.7 (2.36–9.65)		
**Subgroup of patients with vitamin D supplementation**				
Male gender	0.7	0.78 (0.23–2.61)		
Age	0.3	0.92 (0.97–1.02)		
Coffee, cups per day	0.02	0.11 (0.02–0.75)	0.04	0.11 (0.01–0.92)
Presence of diabetes	0.3	1.90 (0.51–7.03)		
MELD score	0.0008	1.24 (1.09–1.41)	0.05	1.16 (1.00–1.35)
Leukocytes / nl	0.2	0.58 (0.59–1.12)		
Hemoglobin (g/dL)	0.003	0.58 (0.41–0.83)		
25(OH)D_3_ <10ng/mL	0.9	0.41 (0.01–9.88)		
Presence/history of ascites	0.01	13.5 (1.65–111)		
**Subgroup of patients without vitamin D supplementation**				
Age	0.1	1.02 (0.97–1.08)		
Coffee, cups per day	0.0001	0.41 (0.14–0.82)	0.003	0.36 (0.19–0.71)
Presence of diabetes	0.006	2.92 (1.35–6.32)	0.006	4.72 (1.56–14.3)
MELD score	0.00001	1.21 (1.11–1.33)	0.007	1.18 (1.04–1.34)
Leukocytes / nl	0.5	0.95 (0.80–1.12)		
Hemoglobin (g/dL)	<0.00001	0.63 (0.52–0.75)	0.002	0.70 (0.56–0.88)
25(OH)D_3_ <10ng/mL	0.02	2.43 (1.14–5.00)	0.04	3.03 (1.06–9.01)
Presence/history of ascites	0.003	4.29 (1.94–9.44)		

MELD, model of end-stage liver disease.

### Vitamin D deficiency is associated with markers of systemic inflammation and bacterial translocation

**Fig 2:**
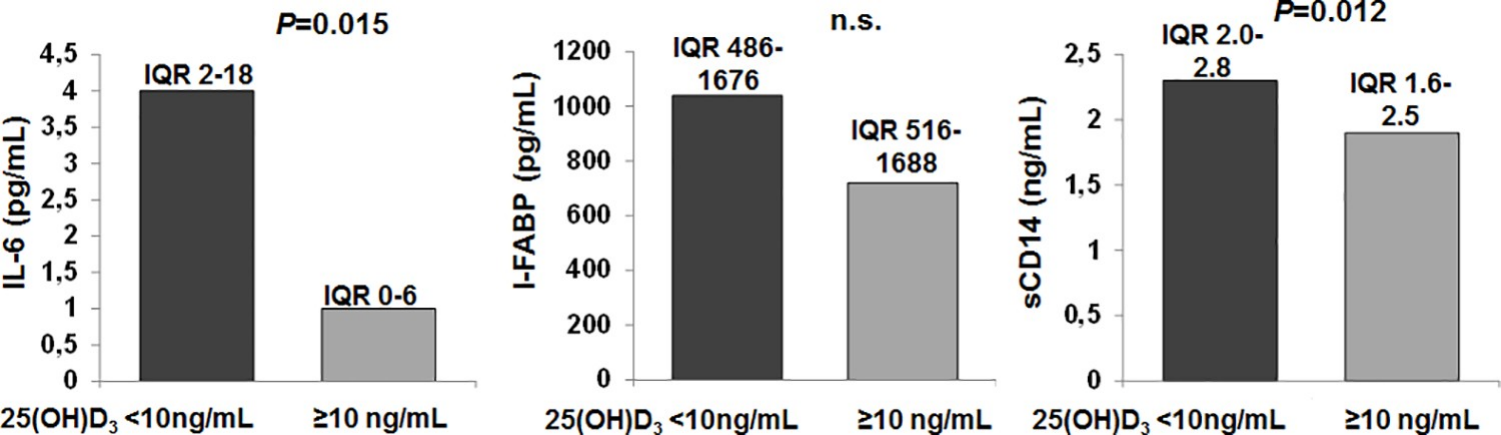
Baseline serum concentrations of IL-6, IFABP, and soluble CD14 accoding to the presence / absence of severe vitamin D deficiency. IQR, interquartile range.

Due to the anti-inflammatory and barrier-strengthening properties of VDR signaling, cirrhosis-associated systemic inflammation and dysfunction of intestinal barriers might be promoted by vitamin D deficiency. Indeed, patients with *versus* without severe vitamin D deficiency (25(OH)D_3_ serum concentration) had significantly higher serum concentrations of CRP (0.85 mg/dL (SD = 1.3) *versus* 0.43 mg/dL (SD = 0.7), P = 0.007). Therefore, we assessed possible associations between 25(OH)D_3_ serum concentration and IL-6, I-FABP, and soluble CD14, as additional surrogate markers of systemic inflammation, intestinal barrier dysfunction, and bacterial translocation, respectively. We performed this analyses in a subgroup of patients for which follow-up serum samples were available and in whom 25(OH)D_3_ serum concentrations remained stable at low levels from baseline to follow-up (n = 39, median 25(OH)D_3_ concentration at baseline = 12 ng/mL and 10 ng/mL at follow-up) or in whom *de novo* supplementation of vitamin D was initiated at baseline (n = 55, median 25(OH)D_3_ concentration at baseline = 9,8 ng/ml and 31 ng/ml at follow-up; P<0.0001). Baseline serum levels of IL-6 and soluble CD14, but not of I-FABP, were significantly higher in patients with severe vitamin D deficiency at baseline compared to patients without severe vitamin D deficiency (Fig 2). During follow-up, soluble CD14 levels declined in comparison to baseline in patients with *de novo* supplementation of vitamin D (median 2.15 ng/mL vs. 1.87 ng/mL, P = 0.002), whereas no change was observed in patients with stably low vitamin D serum levels (median 1.97 ng/mL vs. 2.02 ng/mL, n.s.). However, no significant changes between baseline and follow-up were observed for IL-6 serum levels.

## Discussion

The first main finding of our study is that severe vitamin D deficiency is frequent in patients with decompensated liver cirrhosis and is associated with the risk of hospitalization due to hepatic decompensation and death. Furthermore, our study shows that vitamin D supplementation is effective in patients with advanced liver disease and can achieve serum concentrations of 25(OH)D_3_ in a healthy range. Finally, our study reveals an association between low 25(OH)D_3_ serum levels and liver cirrhosis-associated systemic inflammation, which may provide a functional basis of the relationship between vitamin D deficiency and hepatic decompensation.

Vitamin D deficiency is a frequent finding in healthy world-wide populations [19]. However, the proportion of patients with liver disease suffering from severe vitamin D deficiency is significantly higher compared to healthy controls [17]. An important finding of previous research of our and other groups was that liver-disease associated vitamin D deficiency in non-cirrhotic patients appears to be a result of the underlying liver disease per se, namely infections with hepatotropic viruses or alcoholic and non-alcoholic fatty liver disease, whereas the degree of liver fibrosis has little impact on 25(OH)D_3_ serum levels [10, 12–14,17,20]. This suggests a role of inflammation in the development of vitamin D deficiency, and indeed expression of genes regulating vitamin D metabolism is partially regulated by inflammatory cytokines [3]. In the present study we show that in patients with advanced liver fibrosis or cirrhosis, vitamin D deficiency poorly correlates with the degree of fibrosis / cirrhosis or the MELD score as well. In contrast, there is a strong association between 25(OH)D_3_ serum levels and hepatic decompensation, waist circumference and–interestingly–smoking. There is a clinically established role of vitamin D in maintaining bone health and, importantly, muscular integrity and prevention of frailty [21]. Hence, the high risk of vitamin D deficiency in patients with liver cirrhosis should not be neglected, in particular in a pre-transplant setting where optimal bone density is important due to the risk of post-transplant glucocorticoid therapy and due to the detrimental effect of frailty on post-transplant outcome. Our study firmly shows that adequate 25(OH)D_3_ serum levels can be achieved in patients with advanced liver disease by cholecalciferol supplementation.

Limitations of our study are a not directly assessed the vitamin D substitution compliance, however the profound increase in 25(OH)D_3_ serum levels suggest high adherence. Furthermore possible antibiotic intake was not reliably reported for the entire cohort in this study. Thus a possible effect of concomitant antibiotic therapy on the risk of decompensation cannot be assessed.

Possible beneficial effects of vitamin D signaling on liver disease itself are more controversial due to the lack of appropriate controlled interventional studies. Our study cannot provide sufficient evidence for a causal relationship between vitamin D deficiency and systemic inflammation in patients with liver cirrhosis. The decrease of soluble CD14 levels in patients with adequate response to *de novo* supplementation of vitamin D may be a hint for such a causal relationship, and previous interventional studies e.g. in patients with hemodialysis or with lung tuberculosis have convincingly shown that vitamin D supplementation can suppress pro-inflammatory cytokine secretion [4,5]. Furthermore, animal models, observational studies and interventional studies have shown that vitamin D deficiency promotes the development of inflammatory bowel disease by disrupting the integrity of the intestinal barrier [6,7,22], and VDR signaling appears to be crucial for the development of the intestinal microbiome [9]. Finally, vitamin D suppresses liver fibrosis progression by a genomic feed-back mechanism suppressing the fibrotic response of hepatic stellate cells [23]. Together with the established benefit on bone and muscle health, these data may further support vitamin D supplementation in patients with liver cirrhosis and severe vitamin D deficiency.

In conclusion, our study reveals a possibly functional relevant association between severe vitamin D deficiency, systemic inflammation and risk of hepatic decompensation in patients with advanced liver fibrosis / cirrhosis.

## Supporting information

S1 Table Vitamin D serum levels by seasonal variations. (DOCX)
