# Supplementary material for: Vitamin D deficiency is associated with hepatic decompensation and inflammation in patients with liver cirrhosis: A prospective cohort study
Source: PLoS One. 2018 Nov 8;13(11):e0207162. doi: 10.1371/journal.pone.0207162 (PMC6224127; doi:10.1371/journal.pone.0207162)
Supplement: S1 Table — (DOCX) [file pone.0207162.s001.docx]

| **S1 Table. Vitamin D serum levels by seasonal variations** | | | |  |
| --- | --- | --- | --- | --- |
|  | **August-October** | **November-February** | **March-April** |  |
| **25(OH)D_3_ concentration in patients without supplements; median (IQR)** | | | |  |
| Overall | 22 (13-31) | 13 (7,85-25,5) | 17 (8,7-29) |  |
| Male | 23 (13,5-31,5) | 14 (9,7-25) | 15 (8,65-30) |  |
| Female | 18 (11-28) | 13 (6,5 -29) | 20 (9,1-30) |  |
|  |  |  |  |  |
| **25(OH)D_3_ concentration in patients on supplements; median (IQR)** | | | |  |
| Overall | 36 (25-52) | 40 (29-53) | 45 (27,5-60) |  |
| Male | 28 (22-41) | 39 (28,5-52,5) | 43 (22-58,8) |  |
| Female | 40 (27-55) | 42,5 (29-57,2) | 45,5 (32,5-60,8) |  |
|  |  |  |  |  |
|  | | | | |
